# Relationship of Edentulism, Sleep Disordered Breathing and Cardiovascular Disease: NHANES, 2007-2008

**DOI:** 10.9734/CA/2015/17944

**Published:** 2015-04-27

**Authors:** R. Constance Wiener

**Affiliations:** 1 Department of Dental Practice and Rural Health School of Dentistry, West Virginia University, USA; 2 Department of Epidemiology, School of Public Health, West Virginia University, USA

**Keywords:** Edentulism, cardiovascular disease, NHANES

## Abstract

**Background:**

Edentulism, though declining in younger adults, remains prevalent in the U.S. older adult population. Poorer health outcomes, including cardiovascular outcomes have been associated with edentulism. Sleep disorders are also common in older adults and have been associated with cardiovascular disease. The purpose of this study is to determine if edentulism is associated with cardiovascular disease when sleep disorders are included in the analyses.

**Methods:**

Data from the National Health and Nutrition Examination Survey, 2007-2008 were used in this study. Adjusted logistic regression analyses were performed with cardiovascular disease as the dependent variable and dental status (edentulism, dentate) as the key independent variable and sleep variables introduced as potential confounders.

**Results:**

In multivariable analyses, edentulism was independently associated with cardiovascular disease with an adjusted odds ratio of 2.15 (95% CI: 1.54, 3.00). The model included a sleep summary variable, race, sex, education, smoking status, and drinking status, physical activity, body mass index, conditions or disease count, family poverty index, and insurance status.

**Conclusions:**

Edentulism was associated with cardiovascular disease independent of sleep disordered breathing.

## 1. Introduction

The prevalence of edentulism continues to decline in the U.S. and is expected to reach 2.6% by 2050 [1]. However, it currently remains a public health issue. There were 16.2% of adults 65+ years who reported having had all of their teeth extracted to Behavioral Risk Factor Surveillance System researchers in 2012 [2]. Some states, such as Hawaii and California, have the lowest percentages of older adults who are edentulous(7.0% and 8.7%, respectively), while other states, such as Louisiana and West Virginia, have the highest percentages of older adults with all of their teeth extracted (28.8% and 33.8%, respectively) [2]. These percentages, while alarming, are considerably less than they were in 1999 when 26.2% of adults 65+ years had all of their teeth extracted [2]. West Virginia and Kentucky were the states with the highest percentages in 1999 (44.3% and 44.2%, respectively) [2].

Similarly, cardiovascular disease is a serious public health concern. In 2010, 4.1% of the U.S. population had a myocardial infarction, 4.1% had angina or coronary heart disease, and 2.6% had a stroke [2]. Cardiovascular disease is a complex disease with many ecological, psychosocial and biological factors associated with it.

Researchers have shown an association of edentulism with cardiovascular disease [3,4] and coronary artery plaques [5]. In terms of sleep disordered breathing, researchers indicated a link C-reactive protein, a marker of cardiovascular health, and sleep disordered breathing [6]. And researchers have linked edentulism with sleep disordered breathing, [7]. The researchers theorized decreased retropharyngeal occurs during the sleep of individuals who are edentulous, thereby increasing the risk of sleep disturbance, arterial hypertension and cardiovascular disease [7].

The purpose of this study was to determine if edentulism was associated with cardiovascular disease and sleep disorders using a large, nationally representative study. The rationale is that previous researchers have noted a coincidence of cardiovascular and oral disease [8]. Older adults have a high burden for both conditions [8]. Sleep disordered breathing was investigated in the relationship as a possible additional factor or confounder in the association of edentulism and cardiovascular disease.

## 2. Materials and Methods

The National Health and Nutrition Examination Survey (NHANES) 2007-8 data were used for this study. NHANES study design and methodology is presented in detail at the NHANES website, http://wwwn.cdc.gov/nchs/nhanes/search/nhanes 07_08.aspx. The data were collected from US non-institutionalized in a nationally representative manner using a stratified multistage, probability sample. Physical examinations, laboratory examinations and interview questions were conducted.

The questions asked in the NHANES cardiovascular questionnaire were posed to participants who were age 40 + years. Therefore, the data used for this current study were restricted to data for individuals 40 + years. Additionally, the eligible participants for this study completed the oral examination and had data on the presence or absence of teeth, responded to questions concerning other diseases and conditions, responded to questions concerning family income, and responded to the question concerning their number of hours of sleep per night. The study sample size was 1,525.

The dependent variable was cardiovascular disease. It was identified as at least one positive response to the interview questions of having, or having had congestive heart failure, angina, myocardial infarction, coronary heart disease, or stroke. The main independent variable was edentulism. An oral examination was conducted. Any teeth present was indicated as “dentate” and the absence of all teeth was indicated as “edentulous.” Sleep disordered breathing was indicated through several NHANES questions. “How much sleep do you get (hours)?” was categorized as 5 hours or less, 6 hours, 7 hours, 8 hours and 9 hours or more. “How often do you snore?” was categorized as 0-2 nights/week, 3-4 nights/week and 5 and above. “How often do you snort/stop breathing?” was similarly categorized. “How often do you feel overly sleepy during the day” was categorized as 0-1 times/month, 2-4 times/month, and 5 and above nights per week.

A sleep disordered breathing summary scale of sleep hours, snoring, snorting/stopping breathing, and daytime sleepiness was also used in the analyses [6]. To create the summary, each of the sleep factors were dichotomized: sleep hours was dichotomized at 5 hours or less, and above 5 hours; snoring and snorting/stopping breathing were dichotomized at 0-2 nights/week, and 3 or more nights/week; and daytime sleepiness was dichotomized at 0-4 times/month, and 5 or more times/month. A value of one was assigned if sleep hours were 5 hours or less and a value of zero was assigned to above 5 hours. A value of one was assigned to snoring 3 or more nights/week, and a value of zero was assigned to snorting/stopping breathing fewer than 3 nights/week. A value of one was assigned to snorting/stopping breathing 3 or more nights/week, and a value of zero was assigned to snoring fewer than 3 nights/week. A value of one was assigned to daytime sleepiness of 5 or more times/month and a value of zero was assigned to daytime sleepiness of fewer than 5 times/month. The factors were added for a potential summary score of 0 (no sleep disturbance) to 4 (disturbed sleep).

The additional variables in the analyses included race (non-Hispanic white, non-Hispanic Black, Mexican American and other), sex (male, female), education (less than high school, high school graduate, some college or technical school, college or technical degree and above), smoking status (never, current, former), drinking status (non-drinker, moderate drinker, heavy drinker), physical activity (moderate/vigorous, little/no), body mass index (0 to less than 25, 25 to less than 30, 30 and above), conditions or disease count (0-1, 2 or more), family poverty index (0 to less than 1.25, 1.25 to less than 2.0, 2.0 to less than 4.0, 4.0 and above) and insurance (insured, not insured).

Statistical analyses were conducted with SAS (version 9.3, Cary, NC) and were adjusted for the complex survey design. Frequency of cardiovascular disease with edentulism and the sleep variables were conducted. Crude odds ratios for cardiovascular disease and edentulism, and the sleep variables were analyzed as well as adjusted odds ratios in two models including edentulism in the models. The first model included race, sex, education, smoking status, and drinking status, but did not include physical activity, body mass index, conditions or disease count, family poverty index, and insurance status which was included in the second model. Similarly, crude odds ratios for edentulism and the sleep variables were analyzed as well as adjusted odds ratios in two models.

## 3. Results and Discussion

The sample's baseline characteristics are available in Table 1. There were 55.2% females, 50.4% 55 years and above, 76.5% non-Hispanic white, and 27.8% high school graduates. Most were insured (89.5%), non-smokers (45.0%), moderate drinkers (41.4%) and physically inactive (86.3%). There were 86.7% who were dentate and 72.3% who did not have cardiovascular disease.

Rao Scott Chi Square analyses for the variables with dental status are presented in Table 2. There is a significant relationship of dental status and cardiovascular disease. Other significant findings are with age, race/ethnicity, education, family income ratio, smoking status, alcohol intake, physical activity, and the presence of chronic conditions.

Logistic regression on cardiovascular disease and the percent of people with cardiovascular disease and dental status or sleeping status are presented in Table 3. There were 6.4% of the sample who were edentulous and who also had cardiovascular disease. There were 5.3% of the sample who slept 5 hours or less and who also had cardiovascular disease, 15.39% who snored 3 or more nights/week who also had cardiovascular disease, 6.3% who snorted/stopped breathing, 6.8% who reported 5 or more times/month of daytime sleepiness and while there were 11.6% who snored, and 9.8% who reported a summary score of 2 who also had had cardiovascular disease.

The unadjusted odds ratio for cardiovascular disease and edentulism was 2.86 (95%CI: 2.37, 3.44). The adjusted odds ratio for the first model was 2.32 (95%CI: 1.65, 3.27) and for the second model, it was 2.15 (95%CI: 1.54, 300). These models included controlling for the factors listed in the Methods section as well as the sleep summary score. Edentulism remained independently associated with cardiovascular disease when the sleep variables were considered separately.

The results of the association of edentulism and the sleep variables are presented in Table 4. The unadjusted odds ratio for edentulism for 9 hours or more of sleep was 1.80 (95%CI: 1.03, 3.13). The other relationships did not reach significance.

In this study, edentulism was independently associated with cardiovascular disease in adjusted logistic relations including sleep disordered breathing variables, race, sex, education, smoking status, drinking status, physical activity, body mass index, condition or disease count, family poverty index and insurance status. The adjusted odds ratio for edentulism with the sleep summary score was 2.32 (95%CI: 1.65, 3.27) for Model 1 and 2.15 (95%CI: 1.54, 3.00) for Model 2. There is little research that exists in regard to edentulism and cardiovascular disease to support or disagree with the results of this study which included sleep disordered breathing. However, Watt, et al. [9] indicated that individuals who were edentulous had a higher risk for cardiovascular mortality (HR, 1.49; 95% CI, 1.16,1.92) and stroke-related mortality 2.97 (95% CI, 1.46, 6.05), as did Brown [10] when he reported a cardiovascular risk ratio of 1.2 (95%CI, 1.1-1.4) for participants ages >65 years. Additionally, Elter [11] indicated that individuals who were edentulous had elevated odds of prevalent coronary heart disease (OR 1.8, 95% CI: 1.4, 2.4).

This study is an epidemiological analysis of NHANES data. The biological plausibility that edentulism is associated with cardiovascular disease is that since tooth loss is generally the result of advanced caries with periapical involvement/abcesses, or chronic periodontal disease, it follows that tooth loss is associated with chronic infection in which there is bacteremia, activation of the host inflammatory response resulting in atheromas and related cardiovascular disease independent of other factors [12-14]. (Periodontal disease was indicated as the main cause of tooth extraction at the tooth level with caries/endodontic disease as the main cause at the individual level with over 51% of teeth extracted for periodontal disease, 35.4% for caries and 9.5% for combined periodontal disease and caries [15]. Spivakovsky [16] reported an increased risk of myocardial infarction and tooth loss due to dental infection in support of the explanation. Additionally, fungal infections occur often and chronically in individuals who wear dentures and these infections increase the risk of chronic inflammatory systemic disease with mediators from the cardiovascular system and liver [12]. Research of gene expression in individuals who wear dentures and have fungal infections (Candida albicans infections) indicate changes which result in lowered epithelial barrier integrity, and increased inflammatory response [12]. Additionally, Candida albicans can take advantage of the inflammatory response to bind to the oral tissue [12].

The results of this study are limited in that they were determinations from a cross-sectional study and as a result neither the temporality, nor causality may be attributed to the relationships reported.

The definition for cardiovascular disease was limited to self-reported coronary artery disease, angina, or history of myocardial infarction or stroke; and he self-reports may be subject to recall bias of the participant. The study strengths include the use of a recent, large, nationally representative study which is reliable and valid.

## 4. Conclusion

Edentulism was associated with cardiovascular disease independent of sleep disordered breathing in this large, nationally representative study of non-institutionalized US residents.

## Figures and Tables

**Table 1:** Sample characteristics: NHANES, 2007-8

Characteristics	N (weighted %)
Total Number	1525 (100%)
**Dental Status**	
Edentulous	259 (13.3%)
Dentate	1266 (86.7%)
**Cardiovascular disease**	
Yes, Cardiovascular disease	496 (27.7%)
No, Cardiovascular disease	1029 (72.3%)
**Sex**	
Women	800 (55.2%)
Men 725 (44.8%)
**Age (years)**	
40-54	276 (49.6%)
55 and above	420 (50.4%)
**Race/ethnicity**	
Non-Hispanic Whites	860 (76.5%)
Non-Hispanic Blacks	310 (10.4%)
Mexican Americans	178 (4.9%)
Others 177 (6.2%)
**Education categories**	
Below high school	530 (25.1%)
High school	400 (27.8%)
Some college/technical school	356 (26.4%)
College/technical degree and above	237 (20.6%)
**Family Income Ratio**	
0 to less than 1.25	480 (23.2%)
1.25 to less than 2.00	311 (17.1%)
2.00 to less than 4.00	419 (26.8%)
4.00 and above	315 (30.9%)
**Smoking**	
Never smoker	668 (45.0%)
Former smoker	532 (34.1%)
Current smoker	322 (20.9%)
**Alcohol intake**	
Heavy	213 (19.5%)
Moderate	426 (41.4%)
Non-drinker	492 (39.1%)
**Physical activity**	
Yes	171 (13.7%)
No	1354 (86.3%)
**Body mass index**	
Normal weight	316 (23.1%)
Overweight	479 (32.2%)
Obese	683 (44.7%)
**Chronic Conditions**	
0-1	154 (8.5%)
2 and above	1371 (91.5%)
**Insurance**	
Yes	1323 (89.5%)
No	199 (10.5%)

**Table 2:** Rao Scott Chi Square analyses of variables with dentate or edentulous status, NHANES, 2007-8

Characteristic	Dentate N (weighted %)	Edentulous N (weighted %)	P-value
**Cardiovascular disease**			**<.0001**
Yes, Cardiovascular disease	372 (21.3%)	124 (65.5%)	
No, Cardiovascular disease	894 (6.4%)	135 (6.9%)	
**Sex**			**0.4374**
Women	656 (47.5%)	144 (7.8%)	
Men	610 (39.3%) 1	115 (5.5%)	
**Age**			**<.0001**
40-54	315 (32.9%)	32 (2.6%)	
55 and above	812 (52.6%)	222 (11.9%)	
**Race/ethnicity**			**.0382**
Non-Hispanic Whites	698 (65.8%)	162 (10.7%)	
Non-Hispanic Blacks	248 (8.8%)	62 (1.6%)	
Mexican Americans	168 (4.7%)	10 (0.2%)	
Others	152 (7.4%)	25 (0.7%)	
**Education categories**			**<.0001**
Below high school	398 (19.8%)	132 (5.4%)	
High school	329 (23.5%)	71 (4.3%)	
Some college/technical school	311 (23.4%)	45 (3.0%)	
College/technical degree and above	226 (20.1%)	11 (0.6%)	
**Family income ratio**			**<.0001**
0 to less than 1.25	374 (18.6%)	106 (4.6%)	
1.25 to less than 2.00	239 (13.4%)	72 (3.7%)	
2.00 to less than 4.00	363 (25.8%)	56 (3.0%)	
4.00 and above	290 (28.9%)	25 (2.0%)	
**Smoking**			**<.0001**
Never smoker	596 (41.8%)	72 (3.2%)	
Former smoker	418 (28.0%)	114 (6.1%)	
Current smoker	249 (16.9%)	73 (4.0%)	
**Alcohol intake**			**<.0001**
Heavy	388 (32.2%)	104 (6.9%)	
Moderate	376 (37.8%)	50 (3.6%)	
Non-Drinker	191 (18.0%)	22 (1.5%)	
**Physical Activity**			**<.0001**
Yes	158 (13.3%)	13 (0.5%)	
No	1108 (73.5%)	246 (12.8%)	
**Body Mass Index**			**0.5714**
Normal weight	258 (20.1%)	58 (3.0%)	
Overweight	404 (28.3%)	75 (3.9%)	
Obese	563 (38.3%)	120 (6.4%)	
**Chronic Conditions 0.0147**			
0-1	137 (7.9%)	17 (0.5%)	
2 and above	1129 (78.8%)	242 (12.7%)	
**Insurance 0.5622**			
Yes	1088 (77.5%)	235 (12.0%)	
No	175 (9.2%)	24 (1.3%)	

**Table 3:** Cardiovascular disease, Edentulism and sleep variables

Variables	% with Cardiovascular Disease	Unadjusted Odds Ratio (95% CI)* for cardiovascular disease	Multivariable model 1 Ratio (95% CI)** for cardiovascular disease	Multivariable model 2 Odds Ratio (95% CI)*** for cardiovascular disease
**Dental Status (includes Sleep Summary Score)**				
Edentulism	124 (6.4%)	2.86 (2.37, 3.44)	2.32 (1.65, 3.27)	2.15 (1.54, 3.00)
Dentate	372 (21.3%)	Referent	Referent	Referent
**Sleep hours**				
5 hours or less	106 (5.3%)	1.45 (0.84, 2.52)	1.34 (0.72, 2.49)	1.24 (0.66, 2.33)
6 hours	109 (6.1%)	1.48 (1.13, 1.95)	1.24 (0.80, 1.92)	1.12 (0.72, 1.75)
7 hours	85 (5.4%)	Referent	Referent	Referent
8 hours	128 (7.2%)	1.74 (1.33, 2.27)	1.32 (0.93, 1.86)	1.32 (0.90, 1.94)
9 hours or more	66 (3.6%)	2.77 (1.64, 4.69)	2.72 (1.48, 5.00)	2.56 (1.45, 4.52)
Edentulism			2.33 (1.60, 3.40)	2.13 (1.49, 3.05)
**Snoring (nights/week)**				
0-2	180 (11.6)	Referent	Referent	Referent
3-4	89 (5.4%)	0.76 (0.54, 1.07)	0.83 (0.59, 1.17)	0.84 (0.59, 1.19)
5 and above	162 (9.9%)	0.76 (0.56, 1.03)	0.91 (0.63, 1.32)	0.87 (0.55, 1.37)
Edentulism			2.09 (1.38, 3.17)	1.84 (1.19, 2.86)
**Snorting/stop breathing (nights/week)**				
0-2	339 (20.8%)	Referent	Referent	Referent
3-4	55 (3.2%)	1.39 (0.91, 2.10)	1.54 (0.76, 3.12)	1.38 (0.67, 2.86)
5 and above	45 (3.1%)	1.49 (0.89, 2.49)	1.92 (0.94, 3.94)	2.00 (0.95, 4.21)
Edentulism			2.27 (1.57, 3.29)	2.00 (1.34, 2.97)
**Daytime sleepiness (times/month)**				
0-1	234 (12.7%)	Referent	Referent	Referent
2-4	142 (8.1%)	1.08 (0.80, 1.46)	1.30 (0.79, 2.16)	1.36 (0.78, 2.36)
5 and above	118 (6.8%)	1.10 (0.90, 1.34)	1.39 (0.94, 2.06)	1.29 (0.88, 1.89)
Edentulism			2.25 (1.58, 3.20)	2.05 (1.45, 2.89)
**Sleep summary score**				
0	116 (6.8%)	Referent	Referent	Referent
1	169 (9.8%)	0.85 (0.67, 1.08)	0.94 (0.72, 1.24)	0.91 (0.62, 1.35)
2	124 (6.5%)	0.79 (0.54, 1.14)	0.82 (0.55, 1.23)	0.75 (0.47, 1.20)
3	87 (4.6%)	1.1 5 (0.76, 1.76)	1.51 (0.81, 2.81)	1.34 (0.69, 2.62)
Edentulism			2.32 (1.65, 3.27)	2.15 (1.54, 3.00)

* Separate Unadjusted logistic regression analyses were conducted with edentulism as the only independent variable in the analysis; sleep hours as the only independent variable in the analysis; snoring as the only independent variable in the regression, snorting as the only independent variable in the analysis; daytime sleepiness as the only independent variable in the analysis; and the sleep summary score as the only independent variable in the analysis;

** Model 1 is adjusted for age (40-45, 55 and above), race (Non-Hispanic white, Non-Hispanic black, Mexican American, Others), sex (male, female), education (less than high school, high school graduate, some college or technical school, college or technical degree and above), smoking status (never, current, former), drinking status (non-drinker, moderate drinker, heavy drinker), and insurance (insured, not insured). Each model also included dental status. The dental status model included the sleep summary score;

*** Model 2 is additionally adjusted for physical activity (moderate/vigorous, little/no), body mass index (0 to less than 25, 25 to less than 30, 30 and above), conditions or disease count (0-1, 2 or more), family poverty index (0 to less than 1.25, 1.25 to less than 2, 2 to less than 4, 4 and above), and insurance (insured, not insured).

dx = disease

**Table 4:** Edentulism and sleep variables

Variables	Unadjusted model Odds Ratio (95% CI)* for cardiovascular disease	Multivariable model 1 Ratio (95% CI)** for cardiovascular disease	Multivariable model 2 Odds Ratio (95% CI)*** for cardiovascular disease
**Sleep hours**			
5 hours or less	1.50 (0.93, 2.42)	1.47 (0.74, 2.92)	1.25 (0.55, 2.82)
6 hours	1.68 (1.07, 1.68)	1.31 (0.67, 1.31)	1.23 (0.62, 2.43)
7 hours	Referent	Referent	Referent
8 hours	1.29 (0.85, 1.94)	1.07 (0.58, 1.07)	1.06 (0.50, 2.20)
9 hours or more	1.80 (1.03, 3,13)	1.08 (0.58, 1.08)	1.04 (0.49, 2.20)
**Snoring (nights/week)**			
0-2	Referent	Referent	Referent
3-4	0.82 (0.55, 1.20)	1.04 (0.69, 1.56)	1.13 (0.69, 1.83)
5 and above	0.69 (0.44, 1.09)	0.89 (0.41, 1.62)	0.94 (0.52, 1.71)
**Snorting/stop breathing (nights/week)**			
0-2	Referent	Referent	Referent
3-4	0.89 (0.51, 1.56)	0.77 (0.34, 1.74)	0.70 (0.30, 1.63)
5 and above	0.91 (0.53, 1.57)	0.76 (0.38, 1.50)	0.73 (0.37, 1.43)
**Daytime sleepiness (times/month)**			
0-1	Referent	Referent	Referent
2-4	0.63 (0.40, 1.01)	0.81 (0.43, 1.53)	0.87 (0.47, 1.60)
5 and above	0.71 (0.46, 1.09)	0.66 (0.29, 1.47)	0.60 (0.27, 1.35)
**Sleep summary score**			
0	Referent	Referent	Referent
1	0.91 (0.53, 1.32)	1.04 (0.66, 1.65)	1.18 (0.75, 1.87)
2	1.13 (0.71, 1.80)	1.26 (0.75, 1.26)	1.28 (0.81, 1.98)
3	0.73 (0.52, 1.02)	0.84 (0.46, 1.54)	0.74 (0.43, 1.28)

* Separate Unadjusted logistic regression analyses were conducted with sleep hours as the only independent variable in the analysis; snoring as the only independent variable in the regression, snorting as the only independent variable in the analysis; daytime sleepiness as the only independent variable in the analysis; and the sleep summary score as the only independent variable in the analysis;

** Model 1 is adjusted for age (40-45, 55 and above), race (Non-Hispanic white, Non-Hispanic black, Mexican American, Others), sex (male, female), education (less than high school, high school graduate, some college or technical school, college or technical degree and above), smoking status (never, current, former), drinking status (non-drinker, moderate drinker, heavy drinker), and insurance (insured, not insured);

*** Model 2 is additionally adjusted for physical activity (moderate/vigorous, little/no), body mass index (0 to less than 25, 25 to less than 30, 30 and above), conditions or disease count (0-1, 2 or more), family poverty index (0 to less than 1.25, 1.25 to less than 2, 2 to less than 4, 4 and above), and insurance (insured, not insured)

## Abbreviations

NHANES: National Health and Nutrition Examination Survey
dx: disease
